# Comparative Strengths of Tetrel, Pnicogen, Chalcogen, and Halogen Bonds and Contributing Factors

**DOI:** 10.3390/molecules23071681

**Published:** 2018-07-10

**Authors:** Wenbo Dong, Qingzhong Li, Steve Scheiner

**Affiliations:** 1 The Laboratory of Theoretical and Computational Chemistry, School of Chemistry and Chemical Engineering, Yantai University, Yantai 264005, China; dongwenbo1994@163.com; 2 Department of Chemistry and Biochemistry, Utah State University, Logan, UT 84322-0300, USA

**Keywords:** halogen bond, chalcogen bond, pnicogen bond, tetrel bond

## Abstract

Ab initio calculations are employed to assess the relative strengths of various noncovalent bonds. Tetrel, pnicogen, chalcogen, and halogen atoms are represented by third-row atoms Ge, As, Se, and Br, respectively. Each atom was placed in a series of molecular bonding situations, beginning with all H atoms, then progressing to methyl substitutions, and F substituents placed in various locations around the central atom. Each Lewis acid was allowed to engage in a complex with NH_3_ as a common nucleophile, and the strength and other aspects of the dimer were assessed. In the context of fully hydrogenated acids, the strengths of the various bonds varied in the pattern of chalcogen > halogen > pnicogen ≈ tetrel. Methyl substitution weakened all bonds, but not in a uniform manner, resulting in a greatly weakened halogen bond. Fluorosubstitution strengthened the interactions, increasing its effect as the number of F atoms rises. The effect was strongest when the F atom lay directly opposite the base, resulting in a halogen > chalcogen > pnicogen > tetrel order of bond strength. Replacing third-row atoms by their second-row counterparts weakened the bonds, but not uniformly. Tetrel bonds were weakest for the fully hydrogenated acids and surpassed pnicogen bonds when F had been added to the acid.

## 1. Introduction

A revolution of sorts, albeit a gradual one, occurred in the field of noncovalent interactions as it became progressively more apparent that the venerable H-bond was not completely unique. That is, the bridging proton in H-bonds could be replaced by a variety of other atoms, with little if any loss in noncovalent bond energy. The first class of atoms that fit this criterion was the halogens [1,2,3,4,5,6,7,8,9,10]. The ability of these very electronegative atoms to replace an H atom was deemed counterintuitive at first, as the polarity of the R-H bond, placing a partial positive charge on the proton, was considered a prime ingredient of the classic H-bond. This issue was resolved when it was found that the charge distribution around the halogen atom in an analogous R-X (X = halogen) bond is highly asymmetric. While there is an equatorial band of negative electrostatic potential surrounding the X atom in a R-X bond, there is also a positive polar region situated directly opposite the R atom. This positive area, frequently referred to as a σ-hole, can attract an approaching nucleophile in precisely the same way the partially positive charge surrounding the proton of a H-bond can [11,12]. Of course, the interaction, whether an H or halogen bond, is not entirely electrostatic as it contains other elements, such as charge transfer and dispersion, but this charge distribution voided the argument that a halogen atom must necessarily repel an incoming nucleophile.

As further work proceeded, it soon became apparent that this same phenomenon can be extended to more than just the halogen family of elements. Chalcogen atoms of the S/Se group could engage [13,14,15,16,17,18,19,20,21] in very similar bonding to a nucleophile. As these atoms are commonly involved in covalent bonding with two substituents, one would expect a pair of such σ-holes, one opposite each of these two substituents. Indeed, chalcogen bonding reflected this pattern, as there were two such sites that could engage in these interactions. Along similar lines of thinking, there ought to be three σ-holes surrounding a trivalent pnicogen atom, each of which is in principle capable of forming a so-called pnicogen bond, which has in fact been observed [22,23,24,25,26,27]. Tetrel atoms of the Si/Ge family, too, can participate [28,29,30,31,32,33,34,35,36] in analogously named bonds, and their most common tetravalent covalent bonding situation provides four separate sites for potential tetrel bonds.

These various noncovalent bond analogues of the H-bond share a number of characteristics [37,38,39,40,41,42,43,44,45,46,47,48,49,50,51,52,53,54]. For example, regardless of the particular family of atoms, as one moves down a column of the periodic table, the atom becomes progressively less electronegative and more polarizable. Both of these factors tend to amplify the σ-hole, and reinforce charge transfer from the nucleophile, and to thus strengthen the corresponding noncovalent bond. These same factors can also be intensified if electron-withdrawing substituents are added to the atom in question. Scores of previous results have accordingly demonstrated the ability of such substituents to strengthen the noncovalent bond to the approaching nucleophile. This effect is most pronounced when the substituent lies directly opposite the nucleophile, where it can better accommodate additional charge accumulation from the electron donor. Due to a strong electrostatic component in all of these bonds, they are dramatically strengthened if the Lewis acid is positively charged, with a like reinforcement upon interaction with an anion.

One issue that has borne only very moderate study is the comparison of these different noncovalent bonds with an eye toward those factors that differentiate one from another. While a substantial amount of work has compared each separate sort of bond with the prototype H-bond [55,56,57,58,59,60,61,62,63,64,65,66,67,68,69,70], much less is known about the strength of one with respect to another. On the face of it, one might think that the least electronegative atom is prone to form the most intense σ-holes, and that is in fact what is seen within any given family of atoms, e.g., the halogens or the chalcogens. This premise would lead to the general conclusion that the noncovalent bonding strength ought to increase in the order halogen < chalcogen < pnicogen < tetrel. However, a scan of the literature would suggest this is not the case. As another issue, in order to approach the pertinent atom, the nucleophile must avoid steric and repulsive electrostatic interactions with any of the substituents or lone electron pairs surrounding this central atom. As one proceeds along the halogen-to-tetrel series, the number of lone pairs diminishes while there is an increase in the number of substituents. This trend will also factor into the relative strengths of the different sorts of bonds.

At this juncture, then, there is a need for a thorough and fair comparison of the different sorts of noncovalent bonds. Can one make the general claim that one type will be stronger than another, given like covalent bonding situations? If this is found to be the case, then can one explain this trend on simple chemical grounds? In order to establish any such pattern, it is essential that the calculations be performed at a level that can be considered fully reliable, particularly if the differences in binding strength are small. The present work attempts to answer these questions using high-level ab initio calculations.

## 2. Systems and Theoretical Methods

In order to establish a valid and consistent baseline, focus was first placed on the third row of the periodic table where Br was compared with Se, As, and Ge, as representative of halogen, chalcogen, pnicogen, and tetrel atoms, respectively. Each was placed in a variety of molecular environments, starting with all H substituents as a base point. In the next phase, one H atom was replaced by a methyl group, as a small representative alkyl chain to which many of these atoms would normally be attached. As electron-withdrawing groups are known to amplify each of these sorts of noncovalent bonds, F atoms were added in various modes. This atom could be added in a position directly opposite the nucleophile where it was thought to have the largest amplifying effect. As an alternative, to take advantage of its electron-withdrawing capability, but without its distortion of the σ* antibonding orbital that acts as a sink to electron density transferred from the nucleophile, the F atom could be placed in a peripheral position, viz. bonded to the atom in question, but not directly opposite the nucleophile. In order to assure that the results were not limited to only third-row atoms, additional calculations were carried out with their second-row counterparts. NH_3_ was taken as the universal nucleophilic partner, as its small size and presence of a single lone pair avoided the complications that might result from interactions other than the ones of interest. 

The complexes and monomers were fully optimized using second-order Møller–Plesset perturbation theory (MP2) with the aug-cc-pVTZ basis set [71,72]. Harmonic vibrational frequencies were then computed at the same level in order to verify that the structures obtained correspond to minima with no imaginary frequencies and to obtain vibrational frequencies. Optimization and frequency calculations were carried out using the Gaussian 09 program [73]. Optimized coordinates of monomers and complexes are supplied in the Supplementary Materials section.

Interaction energy (E_int_) and binding energy (E_b_) were used as a measure of the strength of the interactions; they were calculated as the difference between the energy of the complex relative to the monomers in the complex geometry, and the optimized monomers, respectively. E_int_ was also computed with the CCSD(T) (Coupled Cluster with Single and Double Excitations, with Iterative Triples) method for the optimized MP2 structure. Both terms were corrected for basis set superposition error (BSSE) using the counterpoise procedure [74,75] outlined by Boys and Bernardi.

Molecular electrostatic potentials (MEPs) were computed on the 0.001 au electron density contour at the MP2/aug-cc-pVTZ level with the Wave Function Analysis-Surface Analysis-Suite (WFA-SAS) program [76]. The value of the MEP maximum of the σ-hole of the Lewis acid monomer facing the base was evaluated. The Natural Bond Orbital (NBO) method [77] was utilized to extract atomic charges and intermolecular orbital interactions between occupied and empty orbitals using the NBO-3.1 program, included within the Gaussian-09 program. 

The bonding characteristics were analyzed by means of Atoms-in-Molecules (AIM) theory [78]. The relevant bond critical point (BCP) and topological parameters including electron density, Laplacian, and total electron energy density were obtained using the AIM2000 program [79]. To help understand the origin of the binding within each complex, the interaction energy was decomposed into five physically meaningful terms: electrostatic (E^ele^), exchange (E^ex^), repulsion (E^rep^), polarization (E^pol^), and dispersion (E^disp^). This decomposition was performed using the localized molecular orbital-energy decomposition analysis (LMO-EDA) method [80] at the MP2/aug-cc-pVTZ level via the GAMESS program [81].

## 3. Results

### 3.1. Energies and Geometries

**Figure 1:**
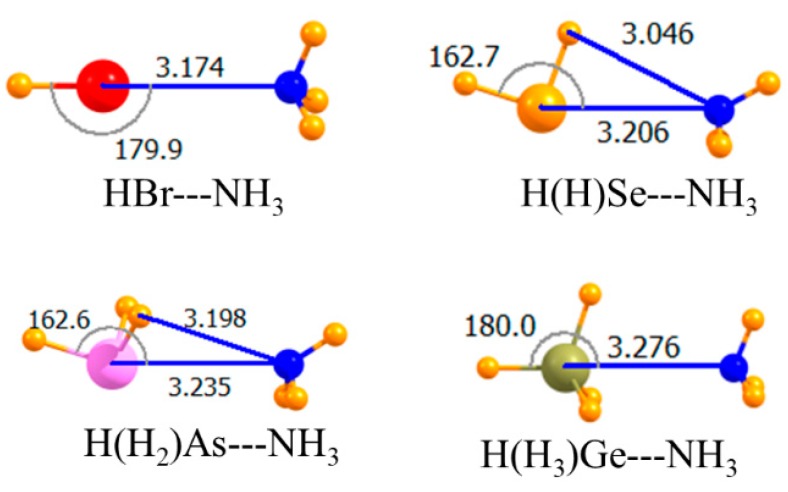
Optimized geometries of complexes of non-fluorinated Lewis acids with NH_3_. Intermolecular distances are in Å, angles in deg.

The diagrams in Figure 1 correspond to the unsubstituted Lewis acids where all atoms bonded to the central A atom are H. As displayed in the topmost section of Table 1, the MP2 interaction energies varied between −6.8 and −9.1 kJ/mol, with the tetrel and chalcogen bonds being the weakest and strongest, respectively. Raising the level of calculation up to CCSD(T) had a small effect, changing these quantities by 0.2–0.4 kJ/mol. This higher-level treatment of correlation weakened all the bonds, with the exception of the tetrel bond, making it slightly stronger than the pnicogen bond. The MP2 binding energies in the third column of Table 1 were only slightly less negative than E_int_, a result of small deformation energies of the two monomers upon forming the complex. The intermolecular R(A∙∙∙N) distances conformed roughly with the energy trends, although the halogen bond was the shortest, despite its being weaker than the chalcogen bond. This may have been partly due to the smaller covalent radius of Br. In terms of mutual orientations, both the halogen and tetrel bonds were fully linear, while the chalcogen and pnicogen bonds deviated by nearly 20° from linearity.

**Figure 2:**
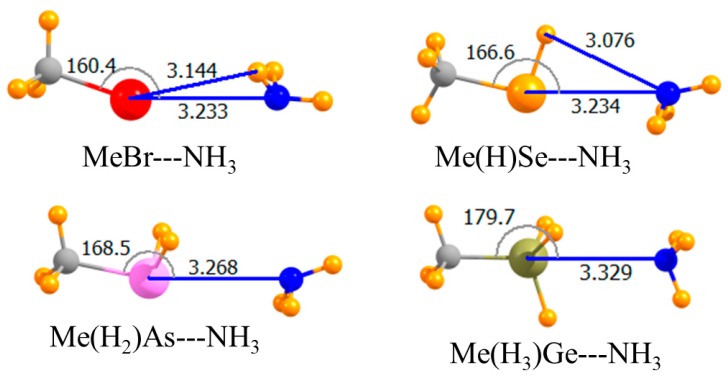
Optimized geometries of complexes of methyl Lewis acids with NH_3_. Intermolecular distances are in Å, angles in deg.

Replacing the H atom that lay opposite the NH_3_ with a methyl group led to the geometries depicted in Figure 2. The only substantive reorientation induced by this methyl substitution involved the Br halogen wherein the NH_3_ came off of the C-Br axis by 20°, and its C_3_ axis turned away from the Br. This methylation weakened all of the bonds, but by varying amounts. This weakening was an expected consequence of the electron-releasing properties of this alkyl group. Using the CCSD(T) data as a reference, the pnicogen bond was weakened by only 0.7 kJ/mol, while the largest decrease of 3.0 kJ/mol occurred for the halogen bond, consistent with its nonlinearity. The chalcogen bond remained the strongest of the four, and it was the halogen bond that was weakest for these methyl-substituted Lewis acids. This same pattern was carried over into the binding energies, which included geometric deformations of the monomers. Commensurate with the weakening of all bonds, the intermolecular distances were all a bit longer for Me-H_n_A. Note also that this substitution induced a 20° nonlinearity into the halogen bond, while slightly enhancing the linearity of the chalcogen and pnicogen bonds.

**Figure 3:**
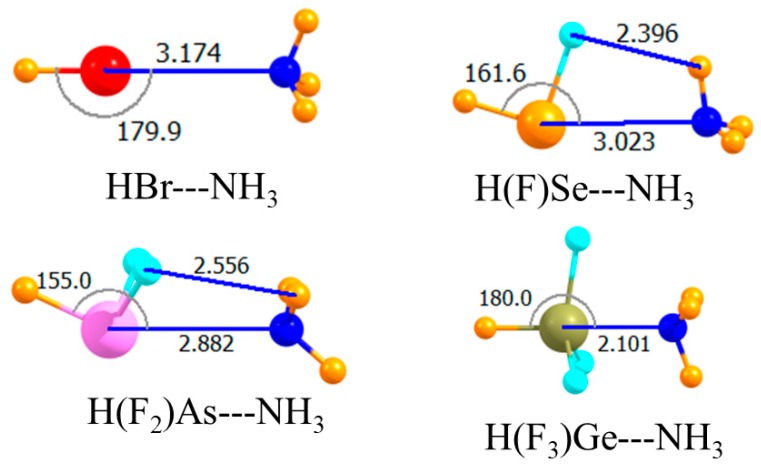
Optimized geometries of complexes involving a partially fluorinated Lewis acid with H opposite N. Intermolecular distances are in Å, angles in deg.

**Figure 4:**
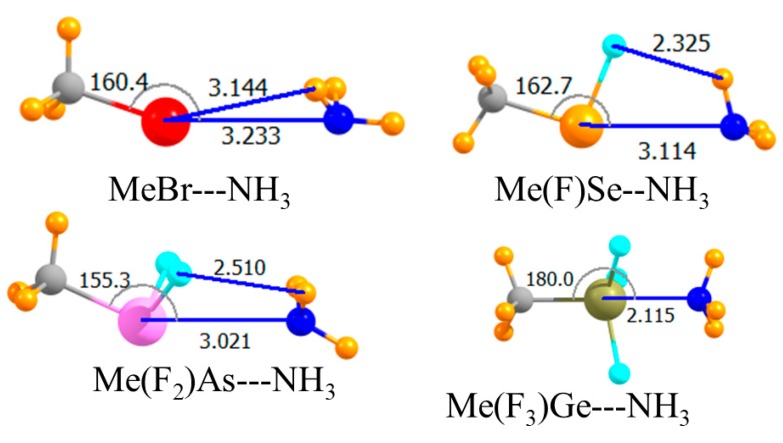
Optimized geometries of complexes involving a fluorinated Lewis acid with CH_3_ opposite N. Intermolecular distances are in Å, angles in deg.

The introduction of electron-withdrawing F atoms onto any given A atom is known to enhance its Lewis acidity. The next section of data in Table 1 relates to replacing all H atoms but the one that lay directly opposite the NH_3_ by F. The molecular structures in Figure 3 indicated very nonlinear chalcogen and pnicogen bonds, with θ(HA···N) equal to 162° and 155°, respectively. Despite this nonlinearity, this substitution substantially increased the strength of both these bonds. The interaction energy of the former rose by 6.6 kJ/mol, and the latter by 12.3 kJ/mol. The larger increment in the case of the pnicogen bond was likely due to the introduction of two F atoms rather than the single F atom for the chalcogen bond. Upon adding a third F atom, there was a dramatic change. The very large increase in the case of the tetrel bond, more than 100 kJ/mol, resulted from its transition into what might be better termed a covalent bond, or at least partially covalent. Note that the R(Ge···N) distance was only 2.1 Å, a contraction of more than a full Å in comparison to the previous two cases. This very close encounter cannot be established without substantial monomer deformation. That is, the three F atoms must have peeled back away from the approaching N as the HGeF_3_ molecule lost its initial pseudo-tetrahedral shape in forming the trigonal bipyramid that encompassed the fifth NH_3_ ligand. This distortion cost some 85 kJ/mol. The final binding energy E_b_ of the tetrel bond was 35 kJ/mol, more than double that of the pnicogen bond, which is itself stronger than the chalcogen bond. As in the earlier case, switching out the H atom on the Lewis acid opposite the NH_3_ with a methyl group, as in Figure 4, weakened all of the interactions. This decrement in the binding energy was only 2–3 kJ/mol for the chalcogen and pnicogen bonds, but amounted to 9 kJ/mol for the tetrel analogue.

**Figure 5:**
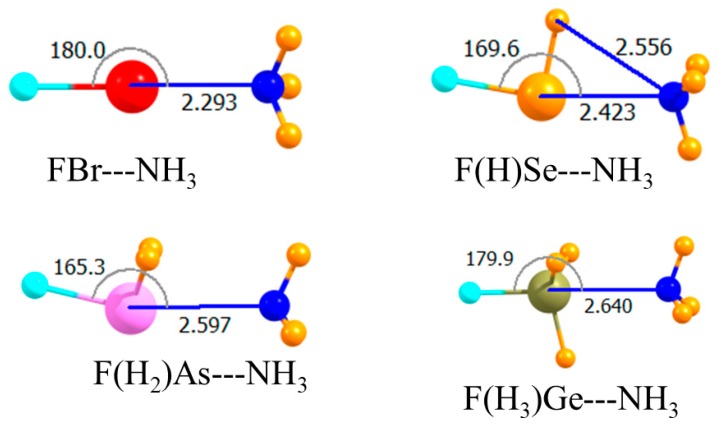
Optimized geometries of complexes involving a partially mono-fluorinated Lewis acid with one F atom opposite N. Intermolecular distances are in Å, angles in deg.

The placement of an electron-withdrawing F atom directly opposite the Lewis base is known to have an even stronger effect than when it is peripherally located. The molecular structures in Figure 5 show again that the halogen and tetrel bonds remained linear, reflecting the symmetry of the Lewis acids, while the less symmetrical chalcogen and pnicogen bonds were substantially distorted from linearity, with the NH_3_ moving up and closer to the H atom(s) of the acid. The bottom section of Table 1 shows the very large binding magnifications quite clearly. Note first that when the F atom was so positioned on the Ge atom, Ge did not engage in a covalent bond with the NH_3_. Indeed, the tetrel bond was the weakest of the array, which followed the pattern halogen > chalcogen > pnicogen > tetrel. This order was a very clear one, with fairly large differences between one bond type and another. Moreover, this pattern was valid not only for E_b_, but for the interaction energy as well. One also saw a clear correlation in that stronger bonds were connected by the shortest intermolecular separations. It might be noted parenthetically that with the F atom opposite the NH_3_, Ge did not engage in a covalent bond with the base, with R(Ge∙∙∙N) = 2.64 Å.

### 3.2. Analysis of the Wave Functions

There are a number of factors that contribute to the strength of noncovalent bonds of this type. As a component of the electrostatic attraction, one typically observes the presence of a so-called σ-hole on the A atom of the Lewis acid. This hole occurs directly opposite one of the covalent bonds in which the A atom is engaged, and attracts the Lewis base. The intensity of this hole is commonly measured by the value of the molecular electrostatic potential (MEP) on a particular isodensity surface, usually taken arbitrarily as ρ = 0.001 au. This maximum, labeled V_s,max_, is reported in the second column of Table 2 for each of the Lewis acid monomers. There are certain points of similarity between these MEP maxima and the interaction energies in Table 1. Taking the F-H_n_A acids as an example, the values of V_s,max_ followed the same halogen > chalcogen > pnicogen > tetrel order as does E_int_, and both exhibited the same (opposite) pattern of tetrel > pnicogen > chalcogen for the H-F_n_A acids. However, there are inconsistencies as well. For example, the replacement of the H atom of HBr by a methyl group reduced the interaction energy while intensifying its σ-hole. Even though H(H_3_)Ge formed the weakest of this class of bonds, it also presented the largest V_s,max_.

In addition to Coulombic attraction between the two monomers, the noncovalent bond depends on a certain amount of intermolecular charge transfer. One way to measure this quantity is as a summation of NBO atomic charges on the atoms of each subunit. This total charge transfer is displayed in the third column of Table 2. Like V_s,max_, CT also correlated generally with the interaction energies. The replacement of one H atom by CH_3_ depressed both quantities, and both were substantially elevated by F-substitution. CT correctly predicted the energetic ordering of the noncovalent bond strengths of the F-H_n_A acids. However, like the MEP maxima, CT failed to correlate with the interaction energies of the non-fluorinated species.

Charge transfer can be understood not only as that between the two molecules as a whole, but also between individual molecular orbitals. The bulk of the charge originates in the N lone pair of NH_3_ that is pointing toward the acid. Its principal sink is the σ* antibonding orbital of the A-R_a_ bond wherein R_a_ lies opposite the N atom. The energetic consequence of this particular charge transfer was calculated by the NBO procedure, and is reported as E(2)^a^ in Table 2. A second portion of the charge originating in the N lone pair made its way into the other σ*(A-R_b_) antibonding orbitals, where R_b_ refers to the peripheral substituents on A, those not opposite the N atom. The cumulative sum of these transfers is tabulated as E(2)^b^ in the fifth column of Table 2. Like the full CT, these individual components only partially reflected the energetics. Methyl substitution correctly reduced E(2), while fluorination led to a marked enhancement. E(2)^a^ followed the same trend, as does the energetics for the F-H_n_A series. However, E(2) was largest for H(H_3_)Ge and smallest for H(H)Se, opposite to the trend in the interaction energies. Clearly, then, while consideration of the MEP and aspects of charge transfer bear some relation to the energetics, neither could be treated as fully predictive. 

On the other hand, this charge transfer into the σ*(A-R_a_) antibonding orbital afforded a reasonable indicator of the properties of this bond. The accumulation of additional charge in this antibonding orbital caused the A-R_a_ bond to weaken, and hence to stretch. This elongation is displayed in the penultimate column of Table 2 and appeared to rise and fall in parallel with E(2)^a^. For example, the longest stretch for most of the acids in the top sections of Table 2 occurred for Ge, as does the largest value of E(2)^a^. The exception to this rule corresponded to those acids in the bottom section of Table 2 where a F atom sat directly opposite the NH_3_ base. In these cases, E(2)^a^ followed the decreasing trend Br > Se > As > Ge, as did Δr. Indeed, there was a tight correlation between these two quantities. The correlation coefficient for a linear relationship between Δr and E(2)^a^ was 0.974, which improved to 0.984 upon eliminating the two Ge systems that engage in a covalent bond.

The last column of Table 2 displays the stretching frequency of this same covalent bond, which shifted to the red in most instances, consistent with its elongation. However, correlations with other parameters were much weaker. For example, even though the charge transfer was quite modest in the complex between H(H)Se and NH_3_, and the binding energy was rather small, the pertinent Se-H bond shifted by a full 108 cm^−1^ to the red, the largest shift of any of these complexes. The A-F red shifts in the F(H_2_)As and F(H_3_)Ge complexes were the reverse of the energetic quantities of these two dimers. One must recall, however, that unlike a particular bond length, the normal vibrational modes did not isolate any one particular bond. Instead they coupled together a number of different bonds, some stretched while others contracted, and included some degree of bending as well. Given this complicated character, it was not surprising to see poor correlation between frequency shifts and other parameters.

The properties of the AIM bond critical point generally offer an accurate barometer of the strength of a noncovalent bond. The three most widely used such quantities are collected in Table 3, and they bore some similarities with energetics. Methyl substitution reduced these values, while they increase upon fluorosubstitution. With respect to the F-H_n_A acids, both ρ and ∇²ρ correctly reproduced the halogen > chalcogen > pnicogen > tetrel energetic trend. However, AIM did not accurately reflect some of the other trends. Taking the unsubstituted H-H_n_A acids as a case in point, neither ρ nor H displayed much differentiation from one type of bond to the next. The values of ∇²ρ were somewhat different from one another, but seemed to exaggerate the strength of the halogen bond.

It is worthwhile to inquire as to how these four sorts of noncovalent bonds compare in terms of their basic contributing factors. Decomposition of the total interaction energy into electrostatic (E^ele^), exchange (E^ex^), repulsion (E^rep^), polarization (E^pol^), and dispersion (E^disp^) provides a fingerprint of sorts for each interaction. These components are listed in Table 4 and show first for the nonfluorinated acids, that the electrostatic attraction was roughly twice that of dispersion, but there was little to distinguish one sort of bond from another in these two elements. On the other hand, exchange was roughly three times larger for the pnicogen and tetrel bonds, as compared to halogen and chalcogen. It was this outsized exchange energy which appeared to be a hallmark of the latter two types of bonds, regardless of substitution. Polarization energy represented the smallest component. With the exception of dispersion, which undergoes a small uptick, all of the other components were lowered upon methyl substitution. Even larger increments accompanied the replacement of one or more H atoms by F. This fluorosubstitution raised the polarization energy to the point where it exceeded dispersion, and could become competitive with the exchange energy for the halogen and chalcogen bonds.

### 3.3. 2nd Row Atoms

It would be injudicious to base all conclusions concerning the comparisons between the various sorts of noncovalent bonds upon atoms in a single row (the third) of the periodic table. Thus, similar calculations were performed for the analogous atoms of the preceding row. The energetic and geometrical data for the complexes of these Lewis acids with NH_3_ are presented in Table 5, which may be directly compared with those in Table 1. It would be expected that the smaller size of the second-row atoms, coupled with their greater electronegativity, ought to have weakened their complexes with NH_3_. This trend was indeed observed, but with some exceptions. For example, the halogen bond of HCl was weaker than that of HBr, but the tetrel bond of H(H_3_)Si was stronger than its third row congener; this same trend was noted after methyl substitution as well. Another issue arises with S. This atom was electronegative enough that it would not engage in a chalcogen bond with NH_3_. Instead, the H_2_S molecule rotated around to form a SH···N H-bond, as does MeSH.

With respect to fluorinated species, the switch from third to second-row atoms produced the expected weakening of the interaction. But another distinction between second and third-row atoms was noted for the fluorinated Me(F_3_)Ge and Me(F_3_)Si acids. Whereas the former pulls in the NH_3_ to form a short covalent Ge-N bond, the same was not true for its Si analogue wherein R(Si···N) remained longer than 3 Å. The trend in binding energies of the F-H_n_A acids remained halogen > chalcogen > pnicogen > tetrel, as it was for the third-row atoms. However, an exception occured in the consideration of the interaction energy. The very large (10 kJ/mol) geometrical distortion energy in FH_3_Si was sufficient to enlarge its interaction energy to exceed that of the pnicogen bond involving FH_2_P. 

While the smaller size of the second-row atoms would tend toward shorter intermolecular separations, the weakening of the interactions should have acted to push the two molecules further apart. The values of R in Table 5 are thus not entirely different from those for the third-row atoms in Table 1. Angular aspects were also generally similar with a few exceptions. The halogen bond of HCl was distorted from linearity by some 23°, and MeCl was also less linear than its MeBr analogue.

## 4. Discussion

In the fully hydrogenated series of Lewis acids, the strengths of the various bonds varied in the pattern chalcogen > halogen > pnicogen > tetrel, although the last two were reversed if the level of calculation was raised from MP2 to CCSD(T). Note that this pattern did not obey the simple order of electronegativity of the central atom, which would have placed the tetrel bond as strongest. Replacement of one H atom by a methyl group (opposite the base) weakened all bonds, but had the strongest effect on the halogen bond, which becomes the weakest of the four. Fluorosubstitution very substantially strengthened the four sorts of bonds as the F atom pulled electron density away from the central atom. Leaving one H atom on the acid to lie opposite the base, and replacing all others by F strengthened the interactions, and this effect rose with the number of these peripheral F atoms. Indeed, the three F atoms of the HF_3_Ge acid enhanced the interaction to the point where the interaction energy exceeded 120 kcal/mol as the very short tetrel bond acquired a covalent character. The very close approach forced the acid to deform to accommodate the base, but even so, the binding energy of the bonds followed the tetrel > pnicogen > chalcogen pattern, opposite to that observed prior to fluorosubstitution. Replacement of the sole H atom by a methyl group again weakened the interactions but leaves the ordering intact. A much more profound strengthening effect occurred if it was the H directly opposite the base that was replaced by F. These interaction energies magnified the unsubstituted interaction energies by a factor between 5 and 9, leading to quantities approaching 70 kcal/mol. It was the halogen bond that was enhanced the most and the tetrel bond the least, so that the order of these monofluorinated bonds washalogen > chalcogen > pnicogen > tetrel. This pattern applied to both interaction and binding energies alike.

The exchange attraction energy was much larger for pnicogen and tetrel bonds than for halogen and chalcogen bonds, making it the largest contributor to the former bond types. With these two exceptions, electrostatics provided the major contribution, surpassing both polarization and dispersion energies by a wide margin. Electrostatic components did not differentiate between the four types of bonds unless there was fluorosubstitution of the Lewis acid. When the F atom was situated directly opposite the base, there was a clear decreasing trend of E^ele^: halogen > chalcogen > pnicogen > tetrel. It was also in these same configurations where polarization energy made a major contribution, but was otherwise generally smaller than the dispersion energy. The value of the molecular electrostatic potential at the site of the sigma hole conformed fairly well to the full electrostatic potential. For example, methyl substitution diminished both E^ele^ and V_s,max_, and both quantities grew upon fluorosubstitution. The intensity of the σ-hole was also a good indicator of E^ele^ in the four sorts of bonds. On the other hand, neither V_s,max_, nor the more complete E^ele^, accurately reproduced trends in the full interaction energy. Charge transfer, either measured as the total from one molecule to the other, or specifically from the N lone pair MO of the base to a σ* antibonding orbital of the acid, also provided some guidance in terms of the full interaction energy. For example, both measures correctly predicted the order of bonding for the fluorinated acids. But there are certain inconsistencies as well in that charge transfer was largest for the tetrel bond, but it was the chalcogen bond that was strongest, when considering unsubstituted systems. Similar limitations applied to the density at the AIM bond critical point, which also showed little distinction between the four sorts of bonds in the absence of F substituents.

There was a general pattern where replacing third-row central atoms with their second-row analogues weakened the various bonds, but this trend was not fully consistent. Taking the non-substituted acids as a case in point, replacement of Br by Cl did substantially weaken the halogen bond. However, there was little effect on the As to P substitution of the pnicogen bond, and the tetrel bond was strengthened when Ge was replaced by Si. There was another issue that must be considered as well. While Se was not electronegative enough to engage in a SeH···N H-bond with NH_3_, the more electronegative S chalcogen atom would form such an H-bond. In fact, the SH···N H-bond was strong enough that it eliminated the S···N chalcogen bond as a secondary minimum on the potential energy surface. The weakening effects of a smaller central atom were more consistently noted, however, when F atoms were added to the Lewis acid. In the case of peripheral substitutions, there was little distinction between chalcogen and pnicogen bonds for the second-row atoms. Whereas the halogen bond remained strongest when F was placed opposite the base, and chalcogen second, there was a sort of reversal in the other two bonds. While the binding energy of the pnicogen bond exceeded that of the tetrel bond, these two reversed when considering only the interaction energy between pre-deformed monomers. This latter issue arose due to the particularly large deformation energy of the FH_3_Si molecule.

There are a number of works in the recent literature that have some bearing on the comparisons of these various sorts of noncovalent bonds. In a general sense, halogen bonding was found [82] preferable to pnicogen bonding when combined with an amine base, but the order reversed for an aromatic π-system. On the other hand, pnicogen bonding is more stable than halogen bonding in dimers formed by HArF and XH_2_P [83]. In broadening the conversation to include chalcogen bonds, Shukla and Chopra [84] investigated how the substituents on the PH_2_R and SeHR molecules determine the structure of the dimer, and thereby the presence of either a pnicogen or chalcogen bond, given the Se···P contact in both. Li and coworkers [85] compared chalcogen with halogen bonds for the fluorosubstituted F_2_C=Se and found the latter to be stronger. However, the comparison was clouded by the different geometries adopted by the two sorts of dimers, one employing a σ-hole and the other a π-hole above the Se atom. 

The Esrafili group has produced some relevant work as well. One study [86] compared chalcogen with pnicogen bonds, both of which could occur, depending upon the molecular orientations, in RHS:PH_2_R dimers. The authors found only small differences, with interaction energies generally in the range between 8 and 18 kJ/mol. Halogen bonds were compared with their pnicogen counterparts [87] in the context of hypervalent ZOF_2_X molecules wherein NH_3_ could interact with either the pnicogen (Z) or halogen (X) atom. A shift from pnicogen to halogen bond preference was observed, consistent with the fact that the halogen bond strengthens as X becomes larger, and the opposite occurs for the X-P···N pnicogen bond. Similar trends were seen in the comparison of halogen with chalcogen bonds for the related YO_2_X_2_ acids [88]. 

Jiao et al. [89] found halogen bonds considerably stronger than pnicogen bonds, but only in a specialized set of dimers, namely dihalogens combined with phosphine derivatives PH_2_R···BrX. Grabowski and Sokalski [36] considered NH_3_ as the Lewis base, along with C_2_H_2_ and Cl^−^. In the case of the former, combined with acids wherein a single F substituent was disposed opposite the base, the order noted for third-row atoms was halogen > chalcogen > pnicogen > tetrel, the same order as obtained here, as are the numerical values. This order persisted for second-row atoms. Shifting gears toward an anionic electron donor, Matile at al [90] have very recently calculated binding energies of various highly fluorinated Lewis acids to Cl^−^, and found pnicogen bonding stronger than chalcogen bonding, and that fourth row atoms engaged in stronger bonds than their third-row analogues. In all cases, it was a C atom (of a phenyl ring) that is situated opposite the base, rather than F. 

There have also been a number of works from this laboratory that relate to the issue of these comparisons. An early set of calculations [91] suggested a pnicogen > halogen > chalcogen bond strength order, but this work was limited to unsubstituted hydrides that were only weakly bound. HSX molecules, capable of both halogen and chalcogen bonds, yielded mixed results depending upon the nature of the X atom [92]. Whether considering a pnicogen, chalcogen, or halogen bond, all display similar sensitivity to stretching [60,93], but greater sensitivity to angular deformation [94], than do H-bonds. However, there is little to distinguish one from another, and all three are subject to similar substituent effects [95], which follow the general pattern CH_3_ < NH_2_ < CF_3_ < OH < Cl < NO_2_ < F. Addition of positive charge on the electron acceptor strengthens all of these interactions, but has more of an effect upon a S···O chalcogen than a C···O tetrel bond, leading to a preference for the former [64]. Within the specialized context of bipodal receptors that engage in a pair of simultaneous noncovalent bonds with a halide [96,97,98], tetrel bonding has a clear edge over halogen, chalcogen, and pnicogen bonds. In another specialized context of hypervalency [99], pnicogen bonds show a clear edge over both chalcogen and halogen bonds. 

## 5. Conclusions

In the absence of any replacements of H atoms, the chalcogen bond is the strongest followed in order by halogen, pnicogen, and tetrel. Methyl substitution on the Lewis acid weakens all bonds, particularly the halogen bond, which is the weakest of the four in this bonding environment. All bonds are strengthened by fluorosubstitution (peripheral to, rather than opposite the base), which leads to the bonding order: tetrel > pnicogen > chalcogen. The most dramatic bond enhancement arises from replacement of the atom opposite the base by F, and yet a different order of halogen > chalcogen > pnicogen > tetrel. If the third-row Lewis acid atoms are replaced by their second-row analogues, there is a general weakening of the noncovalent bonds, but this change is not consistent from one sort of bond to the next.

## Figures and Tables

**Table 1:** Interaction energy (E_int_), binding energy (E_b_), intermolecular distance (R, Å), and angle θ(R-A···N)(deg) where R represents the atom directly opposite NH_3_. Energies are in kJ/mol.

Lewis Acid	E_int,MP2_	E_int,CCSD(T)_	E_b,MP2_	R	θ(R-A···N)
H-H_n_A					
HBr	−7.79	−7.57	−7.77	3.174	179.9
H(H)Se	−9.08	−8.68	−8.98	3.206	162.7
H(H_2_)As	−7.10	−6.81	−7.02	3.235	162.6
H(H_3_)Ge	−6.83	−7.07	−6.42	3.276	180.0
Me-H_n_A					
MeBr	−5.01	−4.57	−4.99	3.233	160.4
Me(H)Se	−7.90	−7.50	−7.82	3.234	166.6
Me(H_2_)As	−6.27	−6.09	−6.19	3.268	168.5
Me(H_3_)Ge	−5.29	−5.71	−4.90	3.329	179.7
H-F_n_A					
H(F)Se	−14.43	−14.08	−13.89	3.023	161.6
H(F_2_)As	−18.35	−18.36	−17.11	2.882	155.0
H(F_3_)Ge	−120.73	−122.35	−35.04	2.101	180.0
Me-F_n_A					
Me(F)Se	−11.86	−11.66	−11.46	3.114	162.7
Me(F_2_)As	−14.44	−14.73	−13.64	3.021	155.3
Me(F_3_)Ge	−111.65	−111.27	−25.77	2.115	180.0
F-H_n_A					
FBr	−67.87	−59.18	−61.71	2.293	180.0
F(H)Se	−49.25	−44.35	−45.97	2.423	169.6
F(H_2_)As	−34.57	−32.30	−32.76	2.597	165.3
F(H_3_)Ge	−30.93	−30.59	−25.28	2.640	179.9

**Table 2:** MEP maximum (V_s,max_) of the σ-hole of the acid monomer facing the base, total charge transfer (CT) from the Lewis base to the acid, NBO values of E(2) from the N lone pair of NH_3_ to σ*(A-R) antibonding orbitals where A is the central atom of the Lewis acid and R is (a) the atom directly opposite N and (b) the peripheral atom(s). Also shown is change in A-R bond length (Δr) and vibrational frequency (∆ν) of the A-R stretch, where R lies opposite N.

Lewis Acid	V_s,max_ au	CT me	E(2) ^a^ kcal/mol	E(2) ^b^ kcal/mol	Δr(A-R) Å	∆ν(A-R) cm^−1^
H-H_n_A						
HBr	0.027	2	11.37	-	0.004	−26.8
H(H)Se	0.030	4	8.99	0.54	0.003	−107.6
H(H_2_)As	0.028	7	10.70	2.51	0.006	−5.4
H(H_3_)Ge	0.032	9	12.37	6.65	0.008	+1.2
Me-H_n_A						
MeBr	0.030	1	3.26	-	0.001	−1.3
Me(H)Se	0.018	2	5.10	0.25	0.001	−0.7
Me(H_2_)As	0.019	4	7.77	1.67	0.003	−2.2
Me(H_3_)Ge	0.024	8	11.87	5.02	0.007	−10.1
H-F_n_A						
H(F)Se	0.036	8	14.00	4.26	0.007	−38.1
H(F_2_)As	0.046	27	15.34	14.46	0.009	−44.8
H(F_3_)Ge	0.069	175	86.19	416.95	0.017	−2.2
Me-F_n_A						
Me(F)Se	0.022	5	8.95	2.63	0.005	−6.7
Me(F_2_)As	0.033	7	8.03	7.65	0.009	−35.7
Me(F_3_)Ge	0.055	172	80.47	417.54	0.021	−101.6
F-H_n_A						
FBr	0.093	143	255.40	-	0.070	−114.9
F(H)Se·	0.089	90	152.82	11.03	0.049	−107.6
F(H_2_)As	0.079	53	82.81	14.96	0.033	−60.2
F(H_3_)Ge	0.077	46	59.11	39.12	0.027	−63.3

^a^ N_lp_→σ*(A-R), R directly opposite N; ^b^ N_lp_→σ*(A-R), R peripheral atom, sum of all such transfer energies.

**Table 3:** Electron density (ρ), Laplacian (∇²ρ), and energy density (H) at the intermolecular BCP in the complexes (all are in au).

Lewis Acid	ρ	∇²ρ	H
H-H_n_A			
HBr	0.010	0.038	0.002
H(H)Se	0.009	0.034	0.001
H(H_2_)As	0.009	0.029	0.001
H(H_3_)Ge	0.008	0.025	0.001
Me-H_n_A			
MeBr	0.008	0.034	0.002
Me(H)Se	0.008	0.032	0.002
Me(H_2_)As	0.008	0.027	0.001
Me(H_3_)Ge	0.007	0.023	0.001
H-F_n_A			
H(F)Se	0.014	0.046	0.001
H(F_2_)As	0.018	0.046	0.000
H(F_3_)Ge	0.077	0.216	−0.030
Me-F_n_A			
Me(F)Se	0.011	0.039	0.002
Me(F_2_)As	0.013	0.037	0.001
Me(F_3_)Ge	0.075	0.209	−0.028
F-H_n_A			
FBr	0.061	0.132	−0.015
F(H)Se·	0.044	0.105	−0.007
F(H_2_)As	0.029	0.074	−0.002
F(H_3_)Ge	0.023	0.072	0.000

**Table 4:** Electrostatic (E^ele^), exchange (E^ex^), repulsion (E^rep^), polarization (E^pol^), dispersion (E^disp^), and interaction energies (E_int_); all are in kJ/mol.

Lewis Acid	E^ele^	E^ex^	E^rep^	E^pol^	E^disp^	E_int_
H-H_n_A						
HBr	−16.30	−10.29	49.53	−4.81	−7.27	−7.90
H(H)Se	−16.85	−10.29	48.61	−4.14	−7.69	−9.11
H(H_2_)As	−16.18	−32.02	52.88	−4.43	−7.40	−7.15
H(H_3_)Ge	−16.80	−32.65	53.63	−4.64	−6.40	−6.86
Me-H_n_A						
MeBr	−8.15	−7.53	35.57	−2.34	−8.78	−5.02
Me(H)Se	−12.79	−9.04	42.39	−3.18	−8.74	−7.86
Me(H_2_)As	−13.00	−28.72	47.23	−3.55	−8.23	−6.31
Me(H_3_)Ge	−13.29	−29.59	48.03	−4.14	−6.40	−5.35
H-F_n_A						
H(F)Se	−34.19	−19.00	93.92	−9.91	−10.66	−14.50
H(F_2_)As	−55.05	−83.77	149.44	−17.56	−11.58	−18.52
H(F_3_)Ge	−363.74	−371.98	782.50	−165.65	−2.17	−121.05
Me-F_n_A						
Me(F)Se	−25.67	−15.70	76.37	−7.44	−10.87	−11.95
Me(F_2_)As	−36.91	−59.31	103.54	−10.41	−11.50	−14.59
Me(F_3_)Ge	−352.88	−369.72	773.13	−158.21	−4.31	−111.94
F-H_n_A						
FBr	−197.84	−97.95	554.14	−120.05	−27.67	−68.09
F(H)Se	−146.89	−75.42	407.59	−74.03	−23.12	−49.49
F(H_2_)As	−98.61	−147.64	269.90	−40.67	−17.85	−34.86
F(H_3_)Ge	−89.20	−125.82	228.60	−31.27	−13.42	−31.10

**Table 5:** Interaction energy (E_int_), binding energy (E_b_), intermolecular distance (R, Å), and angle θ(R-A···N)(deg) where R represents the atom directly opposite N. Energies are in kJ/mol.

Lewis Acid	E_int,MP2_	E_int,CCSD(T)_	E_b,MP2_	R	θ(R-A···N)
H-H_n_A					
HCl	−3.77	−3.73	−3.77	3.254	156.5
H(H)S ^a^	---	---	---	---	---
H(H_2_)P	−7.01	−6.47	−6.94	3.281	166.3
H(H_3_)Si	−8.75	−8.87	−8.05	3.187	180.0
Me-H_n_A					
MeCl	−4.01	−3.81	−3.98	3.409	145.2
Me(H)S ^a^	---	---	---	---	---
Me(H_2_)P	−6.57	−6.42	−6.50	3.294	171.2
Me(H_3_)Si	−6.38	−6.74	−5.77	3.257	180.0
H-F_n_A					
H(F)S	−11.12	−9.11	−8.82	3.176	164.0
H(F_2_)P	−11.41	−11.79	−10.93	3.051	159.0
H(F_3_)Si	−106.23	−108.75	−17.19	2.099	180.0
Me-F_n_A					
Me(F)S	−7.09	−7.29	−6.92	3.315	164.2
Me(F_2_)P	−8.25	−8.84	−7.95	3.247	157.0
Me(F_3_)Si	−12.06	−13.51	−7.57	3.086	179.8
F-H_n_A					
FCl	−53.34	−43.90	−45.78	2.231	180.0
F(H)S	−37.36	−33.14	−34.98	2.435	171.0
F(H_2_)P	−28.34	−26.42	−27.12	2.604	167.7
F(H_3_)Si	−35.26	−34.76	−25.01	2.489	180.0

^a^ Does not form a chalcogen bond.

## Supplementary Materials

The following are available online. Optimized coordinates of complexes and monomers.
