# Supplementary material for: Comparative Strengths of Tetrel, Pnicogen, Chalcogen, and Halogen Bonds and Contributing Factors
Source: Molecules. 2018 Jul 10;23(7):1681. doi: 10.3390/molecules23071681 (PMC6100607; doi:10.3390/molecules23071681)
Supplement: Supplementary file 1 [file molecules-23-01681-s001.pdf]

Optimized coordinates of complexes and monomers

$HBr\cdots NH_3$

N	2.48995600	-0.00025400	0.00005300
H	2.87196400	-0.64562900	-0.68018700
H	2.87200100	-0.26415400	0.89981000
H	2.86615700	0.91352300	-0.22052900
Br	-0.68414800	-0.00010300	0.00003000
H	-2.09463700	0.00165500	-0.00052000

$MeBr\cdots NH_3$

N	-2.94578500	0.09932200	0.00000000
H	-2.65683100	0.63181600	-0.81131100
H	-2.65716000	0.63186100	0.81139900
H	-3.95825400	0.08483600	-0.00017900
Br	0.27673800	-0.15488400	0.00000600
C	2.13681200	0.34627100	-0.00001300
H	2.59524000	-0.06468600	-0.89155000
H	2.19576300	1.42812800	-0.00048700
H	2.59504900	-0.06389300	0.89198700

$FBr\cdots NH_3$

N	-0.00005400	0.01054100	2.15363500
H	0.82571500	-0.45707000	2.50703500
H	-0.81927700	-0.46843500	2.50704900
H	-0.00662300	0.96188300	2.50051800
Br	0.00000500	-0.00067000	-0.13937900
F	0.00004300	-0.00963400	-1.96797800

$H(H)Se\cdots NH_3$

N	-2.50868400	0.05057400	-0.00163600
H	-3.37574400	0.57354300	-0.01684400
H	-2.52966400	-0.52437200	0.83192700
H	-2.53614700	-0.58196100	-0.79219600
H	2.09547300	0.34773100	0.00846200
H	0.25365200	1.33312100	-0.01333800
Se	0.69568300	-0.04417900	-0.00019300

$Me(H)Se\cdots NH_3$

N	2.94530900	0.16147400	-0.00041400
H	3.91530500	0.45221500	0.00484300
H	2.83103900	-0.46530500	-0.78761000
H	2.79853000	-0.39178600	0.83518500
H	0.08550600	1.29257500	-0.04621100
Se	-0.27681700	-0.10968600	-0.00186800

C	-2.19685500	0.18074400	0.00590400
H	-2.50969300	0.68578100	-0.90181800
H	-2.65178900	-0.80526300	0.04365100
H	-2.49313500	0.74633600	0.88294600

F(H)Se···NH_3

N	-2.24446200	0.06778500	0.00006700
H	-2.62035000	0.52884600	0.81996200
H	-2.59573800	-0.88247500	-0.00043900
H	-2.62015100	0.52952900	-0.81953600
H	-0.03051100	1.34450700	-0.00018800
Se	0.17334800	-0.09043000	-0.00002800
F	1.96490400	0.11996800	0.00007600

H(F)Se···NH_3

N	2.37125600	-0.26613800	-0.00121200
H	2.25636500	0.73914500	-0.04952600
H	2.95949300	-0.53732600	-0.77932300
H	2.89120900	-0.46509100	0.84449500
Se	-0.65135200	-0.30158500	-0.00046200
F	-0.05735300	1.35967300	0.00021900
H	-2.04371000	0.14307400	0.00659000

Me(F)Se···NH_3

N	2.79879000	-0.19285500	0.00283700
H	2.50847900	0.77771400	0.00867500
H	3.39039600	-0.32196300	-0.80842200
H	3.38208100	-0.33432400	0.81806300
Se	-0.31098100	-0.35290600	-0.00225900
C	-2.16741600	0.12328600	0.00571700
H	-2.42175800	0.68175500	-0.88987500
H	-2.70577800	-0.82574800	0.00808900
H	-2.41365700	0.68138500	0.90382000
F	0.24961700	1.32780700	-0.00196800

H(H_2)As···NH_3

N	-2.52632800	-0.00136900	0.04515700
H	-3.35995200	-0.02265800	0.61999800
H	-2.58561900	-0.79300900	-0.58388200
H	-2.59152400	0.82834300	-0.53211400
H	2.15712500	0.01239700	0.34288600
H	0.36510900	-1.07301700	0.92614800
H	0.35490500	1.09835400	0.89160700
As	0.70740200	-0.00123700	-0.06002200

$Me(H_2)As\cdots NH_3$

N	-2.97439000	0.00220500	0.12940500
H	-3.90314700	-0.03368200	0.53198100
H	-2.89352700	-0.79076100	-0.49537800
H	-2.93886700	0.82913200	-0.45425300
H	-0.00694600	-1.07348500	0.89877500
H	-0.00973500	1.09774600	0.86579700
As	0.28486300	-0.00255400	-0.10886700
C	2.22966500	0.00413200	0.14075400
H	2.64165400	-0.88406400	-0.33292900
H	2.63907200	0.87849000	-0.36012500
H	2.51374900	0.02069700	1.18837500

$F(H_2)As\cdots NH_3$

N	2.37084000	0.08855500	-0.00064000
H	2.75026500	1.02669000	-0.05449200
H	2.74996100	-0.43101400	-0.78365000
H	2.74605800	-0.33613300	0.83940500
H	0.05153200	0.86477900	-1.09391600
H	0.05178300	0.87780400	1.08328700
As	-0.21726500	-0.12849500	0.00065600
F	-1.97508100	0.17981300	-0.00086800

$H(F_2)As\cdots NH_3$

N	2.27941100	0.00013400	-0.23687900
H	2.37622000	0.81341400	0.35906500
H	3.05920200	0.00157000	-0.88213300
H	2.37739800	-0.81451200	0.35701600
As	-0.60115800	-0.00006700	-0.33519700
F	-0.10694600	-1.30490600	0.70147600
F	-0.10738600	1.30502900	0.70138100
H	-2.00149800	-0.00029300	0.26001300

$Me(F_2)As\cdots NH_3$

N	-2.68887600	-0.00041900	-0.11413000
H	-2.63096800	-0.81458100	0.48571900
H	-3.61054100	0.00290000	-0.53232700
H	-2.62641200	0.81108500	0.48868000
As	0.31715000	0.00020900	-0.41170700
C	2.14020700	-0.00086700	0.21871300
H	2.64496900	-0.89064000	-0.15485300
H	2.64791600	0.88579300	-0.15819400
H	2.13591700	0.00111500	1.30595000

F	-0.16890100	1.29776700	0.64662400
F	-0.16953500	-1.29714900	0.64648400

H(H$_3$)Ge···NH$_3$

N	-2.54140200	0.00034000	-0.00050000
H	-2.92264100	-0.79354400	-0.50039400
H	-2.92851200	0.82988500	-0.43346100
H	-2.91730400	-0.03965500	0.93898300
Ge	0.73504200	0.00002300	-0.00005400
H	0.26226300	1.28174600	-0.64484100
H	0.25978800	-0.08191500	1.43162000
H	0.25982200	-1.19849200	-0.78738000
H	2.25506700	-0.00115000	0.00070600

Me(H$_3$)Ge···NH$_3$

N	-3.00876000	0.00006500	0.00043800
H	-3.35958700	-0.02760300	0.94991600
H	-3.40642000	-0.79721000	-0.48081300
H	-3.40157300	0.82730300	-0.43163200
Ge	0.31983900	-0.00019500	-0.00265100
H	-0.17225200	-1.24548300	-0.71486300
H	-0.17284500	1.23772300	-0.72720500
H	-0.18121500	0.00678800	1.42902600
C	2.26396200	0.00042500	0.00583800
H	2.64307900	0.88570800	0.51244600
H	2.64368700	-0.87936200	0.52146500
H	2.64984400	-0.00462500	-1.01159900

F(H$_3$)Ge···NH$_3$

H	-0.08646400	-0.00489300	1.47489700
H	-0.08400600	1.28072100	-0.73422300
H	-0.08390800	-1.27597200	-0.74261000
F	1.99895800	0.00007400	0.00139000
N	-2.40645400	0.00003300	0.00013800
H	-2.79373600	-0.00380800	-0.93631800
H	-2.78375100	0.81535000	0.46907000
H	-2.78385900	-0.81133900	0.47578300
Ge	0.23344600	-0.00003000	-0.00062700

H(F$_3$)Ge···NH$_3$

N	1.70049700	-0.00254600	-0.00168300
H	2.05384100	0.71520900	0.62034400
H	2.05167100	0.17668800	-0.93522400
H	2.05096700	-0.90122300	0.30894100

Ge	-0.40042700	0.00065300	0.00033200
F	-0.12230600	-0.32622600	1.69728400
F	-0.12149900	1.63333300	-0.56577700
F	-0.12655000	-1.30674500	-1.13089700
H	-1.91311100	0.00301100	0.00160400

$Me(F_3)Ge\cdots NH_3$

N	-1.96050500	0.00025500	-0.00004700
H	-2.31191700	0.39330000	-0.86519900
H	-2.31171600	0.55311800	0.77290200
H	-2.31195200	-0.94547600	0.09230600
Ge	0.15421600	-0.00002700	-0.00002400
C	2.07048300	0.00022600	0.00002700
H	2.43295700	-1.01986600	0.09438100
H	2.43273500	0.42866100	-0.93062000
H	2.43266900	0.59209300	0.83632400
F	-0.14801100	-1.01011500	-1.40585800
F	-0.14801900	1.72260600	-0.17150300

$HCl\cdots NH_3$

N	2.12080200	-0.00003400	0.00844200
H	2.41764100	-0.64113000	-0.71663200
H	2.60409300	-0.27253500	0.85517800
H	2.47285700	0.91389500	-0.24772800
H	-2.44534100	-0.00024100	-0.04037200
Cl	-1.17028600	0.00001500	0.00532100

$MeCl\cdots NH_3$

N	-2.86694000	0.08671300	-0.00043700
H	-3.72983200	0.61585200	-0.00477700
H	-2.33019000	0.38649200	-0.80466900
H	-2.34290900	0.37925700	0.81477500
C	2.09742500	0.43476500	0.00053900
H	1.93559600	1.50659800	0.00889000
H	2.63771600	0.12434600	0.88754000
H	2.63219600	0.13737800	-0.89423800
Cl	0.51067400	-0.37444100	-0.00045300

$FCl\cdots NH_3$

N	0.00000100	-0.00009000	2.01083700
H	0.82113400	-0.47856600	2.35929000
H	-0.82483000	-0.47215000	2.35930700
H	0.00370800	0.95009900	2.35972300
F	0.00000200	-0.00016800	-1.93448100

Cl	-0.00000200	0.00016200	-0.22022700

F(H)S···NH_3

N	-2.13524600	0.02701200	-0.00003400
H	-2.54405000	0.46087800	0.81887900
H	-2.42210600	-0.94428600	-0.00043300
H	-2.54411500	0.46160700	-0.81854100
H	0.07895700	1.20039200	0.00012000
F	1.96142800	0.05152300	-0.00005400
S	0.29532400	-0.11446200	0.00004400

H(F)S···NH_3

N	2.19146900	-0.19205200	-0.00221200
H	1.87478700	0.76913800	-0.03989400
H	2.82670200	-0.32513800	-0.77916900
H	2.73950300	-0.28886900	0.84351800
F	-0.55478900	1.07482600	0.00079800
H	-2.28896100	-0.27468800	0.00854300
S	-0.96870100	-0.51309400	-0.00154300

Me(F)S···NH_3

N	2.80905400	-0.27099300	0.00321500
H	2.34181100	0.62616500	-0.04614700
H	3.55870200	-0.25367600	-0.67679800
H	3.24350800	-0.32617300	0.91592400
C	-2.24180300	-0.08220300	0.03469600
H	-2.55441800	0.48770000	-0.83651600
H	-2.73528200	-1.05621600	0.02206400
H	-2.49692900	0.43548000	0.95579900
F	0.04821900	1.09405100	-0.00471900
S	-0.50024500	-0.46059800	-0.03265900

H(H_2)P··NH_3

N	-2.10726800	-0.00384300	0.05862600
H	-3.02703500	-0.19440800	0.43719300
H	-1.94778300	-0.67727300	-0.68077600
H	-2.15741400	0.90377700	-0.38785700
H	2.55901600	0.02340300	0.15936000
H	0.90350200	-1.00808800	0.84999000
H	0.87638500	1.05459400	0.77880700
P	1.16961400	-0.00500700	-0.10447300

Me(H_2)P···NH_3

N	-2.83408700	0.00484500	0.08483300

H	-3.81248600	-0.13066000	0.30851100
H	-2.58634800	-0.69794600	-0.60094100
H	-2.76170100	0.89805200	-0.38685300
H	0.19069700	-0.99769600	0.86394000
H	0.15406000	1.05922600	0.72961000
C	2.30325100	0.02013500	0.03695500
H	2.71405200	-0.88289500	-0.41147300
H	2.68182300	0.87146500	-0.52629000
H	2.64362400	0.09365800	1.06623300
P	0.45302500	-0.02452900	-0.12388600

$F(H_2)P\cdots NH_3$

N	-2.23663200	-0.00014700	0.03182600
H	-2.71060500	-0.03190000	0.92707700
H	-2.56260900	-0.79379800	-0.50693500
H	-2.56290100	0.82954900	-0.44939400
H	0.07690100	-1.02903100	0.76563300
H	0.07663800	1.02922500	0.76441200
F	1.99189500	0.00030200	0.07253400
P	0.36079600	-0.00038200	-0.15842500

$H(F_2)P\cdots NH_3$

N	-2.19948000	-0.00034700	0.15381000
H	-2.26923900	0.81614100	-0.44195100
H	-3.01869800	-0.00550000	0.74852300
H	-2.26485800	-0.81109700	-0.44988700
F	0.45969000	-1.22032300	-0.47526300
F	0.45871200	1.22016500	-0.47547300
H	2.21090800	0.00063200	0.14227500
P	0.83150800	0.00024500	0.49873300

$Me(F_2)P\cdot\cdot NH_3$

N	2.74859500	0.00059000	-0.17325800
H	2.57806900	0.79396400	0.43272300
H	3.73011100	0.02050300	-0.42038900
H	2.59871200	-0.83029000	0.38598800
C	-2.22207700	-0.00834800	0.02953400
H	-2.71815500	0.87410100	-0.37396900
H	-2.70938100	-0.89659700	-0.37185800
H	-2.28593800	-0.00726200	1.11641200
F	0.01378100	-1.20763900	0.43845600
F	-0.00053700	1.21721700	0.43427700
P	-0.48135400	0.00035600	-0.50586000

$H(H_3)Si\cdots NH_3$

N	2.01246800	0.00001700	0.00000100
H	2.39371700	-0.86571900	0.36171100
H	2.39405000	0.74602000	0.56881600
H	2.39389900	0.11951700	-0.93054400
H	-0.72006900	1.29606200	-0.54112900
H	-0.71990300	-1.11662900	-0.85180300
H	-0.71999700	-0.17933100	1.39296900
H	-2.65981300	-0.00010300	-0.00002000
Si	-1.17494000	0.00000500	-0.00000100

$Me(H_3)Si\cdots NH_3$

N	2.78364900	0.00033100	-0.00003400
H	3.17368600	0.90533400	-0.23401300
H	3.16476100	-0.66272200	-0.66400000
H	3.15303500	-0.25109800	0.90878800
H	0.01170000	-0.89122800	-1.07818500
H	0.01462200	-0.48517500	1.30873300
H	0.01130600	1.37919500	-0.23630300
C	-2.35823700	-0.00067500	0.00139400
H	-2.74600100	0.64928000	0.78553100
H	-2.74831200	0.35257800	-0.95293300
H	-2.74575400	-1.00464900	0.17324900
Si	-0.47322600	0.00073000	-0.00135700

$F(H_3)Si\cdots NH_3$

H	-0.01663500	-0.83342000	-1.17187400
H	-0.01621600	1.43123500	-0.13539700
H	-0.01680600	-0.59873300	1.30756600
F	-1.98016100	0.00025000	-0.00008200
N	2.14979800	0.00006100	-0.00003900
H	2.52557300	0.53998600	0.77085100
H	2.52575300	0.39821700	-0.85276400
H	2.52687500	-0.93702600	0.08181100
Si	-0.33969100	-0.00021000	0.00005800

$H(F_3)Si\cdots NH_3$

N	1.57960200	-0.01190300	-0.00773400
H	1.93597100	-0.87584000	0.38440800
H	1.94996500	0.75720300	0.53875200
H	1.93584400	0.07443000	-0.95271700
F	-0.29767300	-1.30373000	-0.93608500
F	-0.27494300	-0.15727600	1.60076600
F	-0.27373400	1.46708900	-0.66034500

H	-1.98956200	0.01578800	0.01041100
Si	-0.51944900	0.00407000	0.00244600

$Me(F_3)Si\cdots NH_3$

N	2.61597100	0.00008400	-0.00044300
H	2.97753100	-0.05355400	0.94371800
H	3.00147000	0.83813600	-0.41766800
H	2.99931700	-0.78685700	-0.50915600
C	-2.31140300	-0.00066900	0.00520500
H	-2.69030100	-0.91488900	0.45867500
H	-2.69063700	0.84792800	0.57182800
H	-2.69525900	0.06451400	-1.01136500
F	0.05065300	-0.09630400	1.50110900
F	0.04326500	1.35051900	-0.66998000
F	0.04382000	-1.25344700	-0.83719900
Si	-0.47036800	0.00008800	-0.00068100

HBr

Br	0.00000000	0.00000000	0.03907200
H	0.00000000	0.00000000	-1.36753400

MeBr

Br	0.41658400	-0.00000100	-0.00000100
C	-1.50861600	0.00000300	0.00000000
H	-1.84297700	-0.56351500	-0.86258100
H	-1.84288200	-0.46527400	0.91932300
H	-1.84288200	1.02879700	-0.05670800

FBr

Br	0.00000000	0.00000000	0.35966800
F	0.00000000	0.00000000	-1.39870800

H(H)Se

H	1.03556900	-0.95924000	0.00000000
H	-1.03556900	-0.95912000	0.00000000
Se	0.00000000	0.05642200	0.00000000

Me(H)Se

H	0.61897200	1.08368800	0.95676100
H	0.61896700	-1.08367600	0.95677300
As	0.41591000	0.00000000	-0.06366100
C	-1.54021300	0.00000100	0.02321700
H	-1.90574200	0.88168300	-0.49765500
H	-1.90572300	-0.88167600	-0.49768000

H	-1.91021800	-0.00001600	1.04331200

F(H)Se

H	0.50946900	1.40259600	0.00018100
Se	0.35530200	-0.04487800	0.00001700
F	-1.39885900	0.01369700	-0.00008600

H(F)Se

Se	0.03298000	-0.35666200	0.00000000
F	0.03298000	1.39892400	0.00000000
H	-1.41814900	-0.46381400	0.00000000

Me(F)Se

Se	-0.11172500	-0.42629600	0.00000100
C	1.50798500	0.58926300	0.00000500
H	1.57070800	1.19583700	0.89780500
H	2.31071600	-0.14911000	-0.00019600
H	1.57050300	1.19606100	-0.89766200
F	-1.18902000	0.96840900	0.00000000

$H(H_2)As$

H	-0.08026800	1.24620200	0.75889100
H	1.12161500	-0.55641800	0.75570900
H	-1.04177000	-0.69508300	0.75512900
As	0.00001300	0.00016100	-0.06878000

$Me(H_2)As$

H	0.61897200	1.08368800	0.95676100
H	0.61896700	-1.08367600	0.95677300
As	0.41591000	0.00000000	-0.06366100
C	-1.54021300	0.00000100	0.02321700
H	-1.90574200	0.88168300	-0.49765500
H	-1.90572300	-0.88167600	-0.49768000
H	-1.91021800	-0.00001600	1.04331200

$F(H_2)As$

H	-0.54630000	-1.07820800	0.96748000
H	-0.54630000	1.07821200	0.96747600
As	-0.34882300	0.00000000	-0.06525500
F	1.40041700	0.00000000	0.02427100

$H(F_2)As$

As	-0.00007400	-0.39160400	-0.05292200
F	1.29543400	0.75309100	0.01707100

F	-1.29509500	0.75340300	0.01701000
H	-0.00061800	-0.63551100	1.43970700

Me(F_2)As

As	-0.09790700	0.00000000	-0.41926900
C	1.65954800	-0.00039900	0.35291100
H	2.19514000	-0.88898600	0.02337800
H	2.19493600	0.88865500	0.02428600
H	1.55534300	-0.00094400	1.43527500
F	-0.70360300	1.29360000	0.56860800
F	-0.70437100	-1.29319100	0.56866800

H(H_3)Ge

Ge	0.00000100	0.00001200	-0.00003400
H	1.22809400	-0.32197500	-0.82072700
H	-1.24013700	-0.24569300	-0.82896300
H	-0.03140600	-0.88329900	1.22651500
H	0.04341700	1.45059500	0.42427800

Me(H_3)Ge

Ge	-0.38634600	0.00000000	-0.00000300
H	-0.91049400	-1.21938900	-0.73654000
H	-0.91046100	1.24760500	-0.68769000
H	-0.91047000	-0.02819700	1.42430300
C	1.55097700	-0.00000300	0.00000000
H	1.92951000	0.87281300	0.52708600
H	1.92953800	-0.89286300	0.49234600
H	1.92958200	0.02006400	-1.01939500

F(H_3)Ge

H	0.75447700	-1.24100000	-0.74754500
H	0.75443400	1.26909300	-0.69881800
H	0.75553100	-0.02820100	1.44816200
F	-1.41217900	-0.00000800	0.00014900
Ge	0.32641200	0.00000600	-0.00009800

H(F_3)Ge

Ge	-0.00009600	-0.00000200	0.26638900
F	0.80226800	-1.35524500	-0.38107000
F	0.77266000	1.37234100	-0.38106800
F	-1.57484600	-0.01709200	-0.38085500
H	0.00234700	0.00002300	1.76249000

Me(F_3)Ge

Ge	-0.00082100	-0.00000200	-0.00001400
C	1.89398700	-0.00004300	-0.00009800
H	2.25374000	0.82857900	0.60334000
H	2.25371700	-0.93695200	0.41579600
H	2.25372700	0.10823400	-1.01942700
F	-0.67020800	-0.16634100	1.56414700
F	-0.67044200	-1.27136700	-0.92606900
F	-0.67033000	1.43776000	-0.63793100

HCl

H	0.00000000	0.00000000	-1.20392200
Cl	0.00000000	0.00000000	0.07081900

MeCl

C	-1.12460100	0.00000300	-0.00000200
H	-1.46614200	-0.62960700	-0.81364800
H	-1.46615000	-0.38985500	0.95207000
H	-1.46614900	1.01944700	-0.13841700
Cl	0.65565000	0.00000000	0.00000000

FCl

F	0.00000000	0.00000000	-1.07141000
Cl	0.00000000	0.00000000	0.56721700

F(H)S

H	-1.28142000	-0.69442200	0.00000000
F	0.05125700	1.07336900	0.00000000
S	0.05125700	-0.56036900	0.00000000

H(F)S

H	-1.28142000	-0.69442200	0.00000000
F	0.05125700	1.07336900	0.00000000
S	0.05125700	-0.56036900	0.00000000

Me(F)S

C	1.34402200	0.41191800	-0.00000600
H	1.39436100	1.02297100	0.89731400
H	2.17361500	-0.29759800	0.00006100
H	1.39444400	1.02298200	-0.89730900
F	-1.23262300	0.60337600	0.00000200
S	-0.12080900	-0.60314100	-0.00000300

$H(H_2)P$

H	-0.10628700	1.18461000	0.63397800

H	1.07934400	-0.50045900	0.63400400
H	-0.97335100	-0.68431300	0.63377800
P	0.00002000	0.00001100	-0.12678400

Me(H_2)P

H	0.92872000	1.02939500	0.80835400
H	0.92874200	-1.02938000	0.80836700
C	-1.18769800	-0.00000200	0.02541500
H	-1.57582000	0.87949800	-0.48519000
H	-1.57584400	-0.87946100	-0.48523900
H	-1.53644000	-0.00002500	1.05416300
P	0.66378900	-0.00000100	-0.12353000

F(H_2)P

H	-0.82065100	-1.02054800	0.81246700
H	-0.82065000	1.02054900	0.81246600
F	1.07740000	0.00000000	0.03172000
P	-0.53702000	0.00000000	-0.12736100

H(F_2)P

F	-1.21666800	-0.49632500	0.02675800
F	1.21665700	-0.49633500	0.02675900
H	-0.00000200	0.93107100	1.24772500
P	0.00000700	0.53352500	-0.11529200

Me(F_2)P

C	-1.54173000	-0.00023400	0.20754900
H	-2.07966500	0.88585400	-0.12791000
H	-2.07977300	-0.88607000	-0.12837200
H	-1.46279900	-0.00049900	1.29348400
F	0.73019100	-1.21202600	0.32035200
F	0.72973200	1.21226600	0.32033200
P	0.11555400	-0.00000300	-0.53657700

H(H_3)Si

H	1.21309200	-0.11689000	-0.83600300
H	-1.19961400	-0.18370900	-0.84340400
H	0.02549600	-1.03713200	1.05253600
H	-0.03897800	1.33762700	0.62719900
Si	0.00000000	0.00000700	-0.00002300

Me(H_3)Si

H	-1.15558800	1.38600600	-0.04795900
H	-1.15561300	-0.65146000	1.22429000

H	-1.15562100	-0.73453100	-1.17632700
C	1.24405100	-0.00000300	-0.00000100
H	1.62876400	-1.01844900	0.03524300
H	1.62874400	0.47871300	-0.89962400
H	1.62873100	0.53975200	0.86438700
Si	-0.63455200	-0.00000100	0.00000000

$F(H_3)Si$

H	0.96570800	-1.20651300	-0.71044800
H	0.96567300	1.21938900	-0.68814500
H	0.96724000	-0.01289700	1.39941100
F	-1.10920900	-0.00000500	0.00017500
Si	0.50601800	0.00000500	-0.00017100

$H(F_3)Si$

F	-1.28200000	-0.74616400	-0.23496100
F	1.28773300	-0.73623200	-0.23496500
F	-0.00574000	1.48301400	-0.23454100
H	0.00001900	-0.00173200	1.77954100
Si	0.00000300	-0.00027300	0.32576200

$Me(F_3)Si$

C	-1.79876500	-0.00000200	0.00004400
H	-2.17741300	0.24704500	-0.99004200
H	-2.17740400	-0.98096700	0.28113800
H	-2.17741000	0.73391400	0.70903600
F	0.62352000	-1.06328600	-1.02710800
F	0.62360500	-0.35784500	1.43436000
F	0.62353900	1.42113200	-0.40730200
Si	0.03491700	0.00000100	0.00000400

NH_3

N	0.00000000	0.00000000	0.11402800
H	0.00000000	0.93804400	-0.26606600
H	-0.81237000	-0.46902200	-0.26606600
H	0.81237000	-0.46902200	-0.26606600
